# Characterization and Genomic Analysis of ValSw3-3, a New *Siphoviridae* Bacteriophage Infecting Vibrio alginolyticus

**DOI:** 10.1128/JVI.00066-20

**Published:** 2020-05-04

**Authors:** Ling Chen, Quan Liu, Jiqiang Fan, Tingwei Yan, Haoran Zhang, Jinfang Yang, Deng Deng, Chaolan Liu, Ting Wei, Yingfei Ma

**Affiliations:** a Shenzhen Institute of Synthetic Biology, Shenzhen Institutes of Advanced Technology, Chinese Academy of Sciences, Shenzhen, China; b Key Laboratory of Quantitative Engineering Biology, Shenzhen Institutes of Advanced Technology, Chinese Academy of Sciences, Shenzhen, China; c Shenzhen Key Laboratory of Synthetic Genomics, Shenzhen Institutes of Advanced Technology, Chinese Academy of Sciences, Shenzhen, China; d Guangdong Provincial Key Laboratory of Synthetic Genomics, Shenzhen, China; e College of Life Science and Oceanography, Shenzhen University, Shenzhen, China; f College of Life Science and Technology, Jinan University, Guangzhou, China; g R&D Center, Shenzhen Alpha Feed Co., Ltd., Shenzhen, China; h Antibiotics Research and Reevaluation Key Laboratory of Sichuan Province, Sichuan Industrial Institute of Antibiotics, Chengdu University, Chengdu, China; University of Texas Southwestern Medical Center

**Keywords:** *Siphoviridae*, *Vibrio alginolyticus*, bacteriophage, genomic analysis

## Abstract

A novel lytic bacteriophage, ValSw3-3, which efficiently infects pathogenic strains of Vibrio alginolyticus, was isolated from sewage water and characterized by microbiological and *in silico* genomic analyses. Transmission electron microscopy indicated that ValSw3-3 has the morphology of siphoviruses. This phage can infect four species in the *Vibrio* genus and has a latent period of 15 min and a burst size of 95 ± 2 PFU/infected bacterium. Genome sequencing results show that ValSw3-3 has a 39,846-bp double-stranded DNA genome with a GC content of 43.1%. The similarity between the genome sequences of ValSw3-3 and those of other phages recorded in the GenBank database was below 50% (42%), suggesting that ValSw3-3 significantly differs from previously reported phages at the DNA level. Multiple genome comparisons and phylogenetic analysis based on the major capsid protein revealed that phage ValSw3-3 is grouped in a clade with five other phages, including *Listonella* phage phiHSIC (GenBank accession no. NC_006953.1), *Vibrio* phage P23 (MK097141.1), *Vibrio* phage pYD8-B (NC_021561.1), *Vibrio* phage 2E1 (KX507045.1), and *Vibrio* phage 12G5 (HQ632860.1), and is distinct from all known genera within the *Siphoviridae* family that have been ratified by the International Committee on Taxonomy of Viruses (ICTV). An *in silico* proteomic comparison of diverse phages from the *Siphoviridae* family supported this clustering result and suggested that ValSw3-3, phiHSIC, P23, pYD8-B, 2E1, and 12G5 should be classified as a novel genus cluster of *Siphoviridae*. A subsequent analysis of core genes also revealed the common genes shared within this new cluster. Overall, these results provide a characterization of *Vibrio* phage ValSw3-3 and support our proposal of a new viral genus within the family *Siphoviridae*.

**IMPORTANCE** Phage therapy has been considered a potential alternative to antibiotic therapy in treating bacterial infections. For controlling the vibriosis-causing pathogen Vibrio alginolyticus, well-documented phage candidates are still lacking. Here, we characterize a novel lytic *Vibrio* phage, ValSw3-3, based on its morphology, host range and infectivity, growth characteristics, stability under various conditions, and genomic features. Our results show that ValSw3-3 could be a potent candidate for phage therapy to treat V. alginolyticus infections due to its stronger infectivity and better pH and thermal stability than those of previously reported *Vibrio* phages. Moreover, genome sequence alignments, phylogenetic analysis, *in silico* proteomic comparison, and core gene analysis all support that this novel phage, ValSw3-3, and five unclassified phages form a clade distant from those of other known genera ratified by the ICTV. Thus, we propose a new viral genus within the *Siphoviridae* family to accommodate this clade, with ValSw3-3 as a representative member.

## Abstract (precis)

Phage therapy has been considered a potential alternative to antibiotic therapy in treating bacterial infections. For controlling the vibriosis-causing pathogen Vibrio alginolyticus, well-documented phage candidates are still lacking. Here, we characterize a novel lytic *Vibrio* phage, ValSw3-3, based on its morphology, host range and infectivity, growth characteristics, stability under various conditions, and genomic features. Our results show that ValSw3-3 could be a potent candidate for phage therapy to treat V. alginolyticus infections due to its stronger infectivity and better pH and thermal stability than those of previously reported *Vibrio* phages. Moreover, genome sequence alignments, phylogenetic analysis, *in silico* proteomic comparison, and core gene analysis all support that this novel phage, ValSw3-3, and five unclassified phages form a clade distant from those of other known genera ratified by the ICTV. Thus, we propose a new viral genus within the *Siphoviridae* family to accommodate this clade, with ValSw3-3 as a representative member.

## INTRODUCTION

Vibrio alginolyticus is one of the most important opportunistic pathogens that can infect a variety of aquatic animals, and it has been implicated in several mass mortality cases in major aquaculture species, from fish to mollusks and crustaceans (1–3). This bacterium is also pathogenic to humans, causing skin and ear infections and acute gastroenteritis (4). Moreover, multidrug-resistant *Vibrio* strains are quickly emerging because of the overuse of antibiotics, and alternative approaches must be developed to control these pathogens. In this context, bacteriophages (or phages) have been considered potential therapeutic agents for use in the treatment of pathogenic *Vibrio* infections in aquaculture (5, 6). Due to their high specificity for host bacteria and ability to proliferate, phages could be a potent and safe biocontrol agent.

For applications in biocontrol, a full characterization of phage genomes is required to rule out the presence of potentially harmful genes related to virulence or antibiotic resistance and to confirm their lytic or temperate nature (7). To date, approximately 300 phages infecting *Vibrio* hosts have been isolated, completely sequenced, and deposited in the National Center for Biotechnology Information (NCBI) genome database (as of 13 June 2019). Most of them are phages infecting V. cholerae, V. parahaemolyticus, and V. vulnificus. Until recently, the complete genomes of only a few V. alginolyticus phages had been reported, including those of four myoviruses (VP01 [1], PVA1 [8], phi-A318 [9], and pVa-21 [10]) and five podoviruses (ValKK3 [11], VEN [7], ΦA318 [9], Φas51 [9], and IME 271 [12]). Lytic phage pVa-21 was isolated from seawater and showed potential as a biocontrol agent against V. alginolyticus because of its anti-planktonic and anti-biofilm activities (10). In addition, V. alginolyticus phage VP01 exhibited therapeutic potential by effectively suppressing the growth of V. alginolyticus (1). Both phages belong to the *Myoviridae* family. On the other hand, the podovirus VEN can exclusively infect its original host, V. alginolyticus strain V2, but is unable to lyse other tested V. alginolyticus strains (1). Interestingly, no siphoviruses isolated using V. alginolyticus as the host strain have been reported with their complete genomic sequences in the literature.

In this study, we report the biological and genomic characterization of a novel lytic siphovirus, named ValSw3-3. This phage was isolated using a pathogenic V. alginolyticus strain from diseased shrimps as the host. To explore the novelty of this phage, phylogenetic analysis based on the major capsid protein and the terminase large subunit, genomic, comparative *in silico* proteomic analyses, and core gene analysis of ValSw3-3 and other related phages were carried out.

## RESULTS AND DISCUSSION

### Biological features of ValSw3-3.

**FIG 1:**
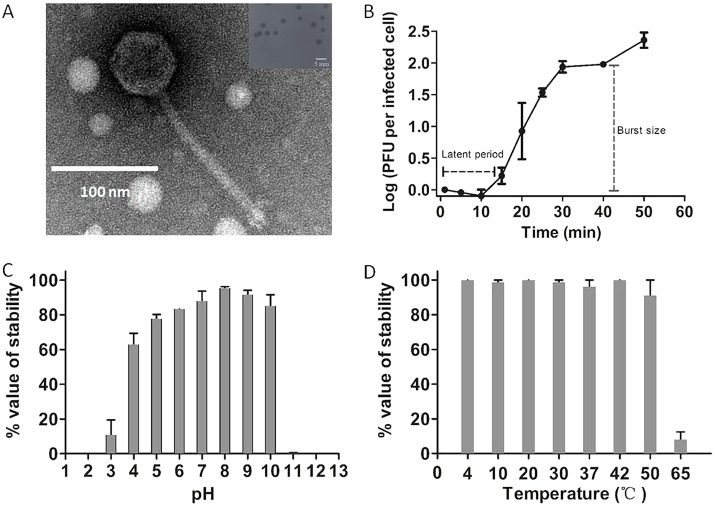
Morphology and biological properties of phage ValSw3-3. (A) Morphology revealed by transmission electron microscopy. Scale bar represents 100 nm. The inset shows plaques of ValSw3-3 formed on the bacterial lawn of a Vibrio alginolyticus strain (GenBank accession no. MH298559), with the scale bar representing 5 mm. The plates were incubated at 30°C, and clear and well-defined plaques were observed and photographed after 12 h. (B) One-step growth curve. Phages were grown in an exponential-phase culture of a V. alginolyticus strain (MH298559). Data points show the numbers of PFU per infected cell in the cultures at different time points. (C) pH stability of ValSw3-3. Phages were incubated for 1 h at different pH values. (D) Thermal stability of ValSw3-3. Phages were incubated for 1 h at different temperatures. Phage titers were determined using the double-layer agar method. Data points represent the mean values ± standard deviations (SD) from three replicate experiments.

By propagating ValSw3-3 on its host strain, Va-F4, plaques were formed on the bacterial lawn with a clear round morphology and a well-defined boundary (Fig. 1A, inset). The plaque size was approximately 2.24 ± 0.14 mm in diameter after 12 h of incubation at 30°C. The morphology of the phage particles observed using transmission electron microscopy (TEM) showed that ValSw3-3 had an icosahedral head (67 ± 4.0 nm) and a noncontractile sheathed tail (168 ± 2.2 nm) (Fig. 1A), indicating that this phage belongs to the family *Siphoviridae* within the *Caudovirales* order.

The one-step growth curve of ValSw3-3 propagated on Va-F4 was plotted and is shown in Fig. 1B. The latent period of phage ValSw3-3 was approximately 15 min. The burst size was 95 ± 2 PFU/cell (burst size is the total number of phages liberated at the end of one cycle of growth/number of infected bacteria). Although the burst size was not significantly higher, the latent period of ValSw3-3 was shorter than those of most previously reported *Vibrio* phages (1, 13, 14).

Spot tests showed that phage ValSw3-3 could infect multiple *Vibrio* species. Among the 34 strains (aside from Va-F4) tested in this study, phage ValSw3-3 was able to infect strains, including V. alginolyticus (Va-F10, Va-F2, and Va-F3), *Vibrio* species (Vs-F1, Vs-6, and Vs-9), V. parahaemolyticus (Vp-F7), and V. metschnikovii (Vm-4) (Table 1). As expected, phage ValSw3-3 did not show infectivity in bacterial strains that do not belong to the *Vibrio* genus. The ability of ValSw3-3 to lyse multiple *Vibrio* strains highlighted that this phage could be explored for phage therapies against vibriosis caused by different *Vibrio* strains. Since ValSw3-3 has a broad host range spanning several species of *Vibrio*, determining the genes encoding tail fibers and other host specificity-related proteins in ValSw3-3 would be valuable.

**TABLE 1:** Host range of phage ValSw3-3

Bacterial species	Infectivity^*d*^			
	Strain	Accession no.	ValSw3-3 lysis	Source or reference
Aeromonas hydrophila	Ah-X6	MH298555	−	Shrimp^*a*^
*Aeromonas* sp.	As-15	MH298572	−	Water^*b*^
Bacillus cereus	Bc-X3	MH298576	−	Shrimp^*a*^
*Bacillus* sp.	Bs-X41	MH298554	−	Shrimp^*a*^
Enterococcus faecalis	Ef-X22	MH298551	−	Shrimp^*a*^
Enterococcus faecalis	Ef-X24	MH298552	−	Shrimp^*a*^
Enterococcus faecalis	Ef-X26	MH298553	−	Shrimp^*a*^
Photobacterium damselae subsp. *damselae*	Pd-D7	MH298546	−	Shrimp^*a*^
Vibrio alginolyticus^*e*^	Va-F4	MH298559	+	36
Vibrio alginolyticus	Va-F3	MH298558	+	36
Vibrio alginolyticus	Va-F10	MH298564	+	36
Vibrio alginolyticus	Va-F2	MH298557	+	36
Vibrio alginolyticus	Va-F12	MH298566	−	Shrimp^*c*^
Vibrio alginolyticus	Va-X15	MH298577	−	Shrimp^*a*^
Vibrio azureus	Va-F9	MH298563	−	36
Vibrio cincinnatiensis	Vc-3	MH298567	−	Water^*b*^
Vibrio metschnikovii	Vm-4	MH298568	+	Water^*b*^
Vibrio metschnikovii	Vm-8	MH298570	−	36
Vibrio natriegens	Vn-19	MH298575	−	36
Vibrio parahaemolyticus	Vp-F7	MH298562	+	36
Vibrio parahaemolyticus	Vp-D3	MH298543	−	36
Vibrio parahaemolyticus	Vp-D4	MH298544	−	36
Vibrio parahaemolyticus	Vp-D5	MH298545	−	36
Vibrio parahaemolyticus	Vp-D9	MH298547	−	36
Vibrio parahaemolyticus	Vp-X10	MH298548	−	36
Vibrio parahaemolyticus	Vp-X11	MH298549	−	36
*Vibrio* sp.	Vs-F5	MH298560	+	Water^*b*^
*Vibrio* sp.	Vs-6	MH298569	+	36
*Vibrio* sp.	Vs-9	MH298571	+	Water^*b*^
*Vibrio* sp.	Vs-17	MH298573	−	36
*Vibrio* sp.	Vs-18	MH298574	−	Water^*b*^
*Vibrio* sp.	Vs-F1	MH298556	−	36
*Vibrio* sp.	Vs-F11	MH298565	−	Shrimp^*c*^
*Vibrio* sp.	Vs-F6	MH298561	−	Shrimp^*c*^
*Vibrio* sp.	Vs-X12	MH298550	−	Shrimp^*a*^

a Strains isolated from the gut of sick shrimp in aquaculture (Shanwei, Guangdong Province, China).

b Strains isolated from water in the Yangzi river (Wuhan, Hubei Province, China).

c Strains isolated from the gut of sick shrimp in aquaculture (Shenzhen, Guangdong Province, China).

d A total of 35 strains were tested during the host range analysis. The ability of ValSw3-3 to infect different *Vibrio* strains was evaluated on the basis of the formation of a lysis-cleared zone at a spot on a bacterial lawn classified into two categories, lysis (+) and no lysis (−).

e The host strain used for ValSw3-3 isolation.

Furthermore, we tested the impacts of pH and temperature on the stability of ValSw3-3, which was estimated by determining the changes in the number of plaque-forming units (PFU). Plaque counting showed that ValSw3-3 displayed a broad stability range (pH 3 to 10) and was observably stable in lytic capacity, with its infectivity maintained above 80% at pH 6 to 10 (Fig. 1C). Meanwhile, short-term thermal stability tests showed that ValSw3-3 was stable at 4°C, 10°C, 20°C, 30°C, 37°C, 42°C, and 50°C (inactivation rate lower than 10%) for 1 h. Although ValSw3-3 was not resistant to even higher temperatures (less than 10% viable at 65°C) (Fig. 1D), it still showed better thermal stability than other reported V. alginolyticus phages, including pVco-14 (50°C), pVa-21 (50°C) (10), and IME271 (40°C) (12), making it suitable for use under normal seawater conditions or extreme environments (i.e., 4 to 50°C and pH 4 to 10).

### *In vitro* lytic effect.

**FIG 2:**
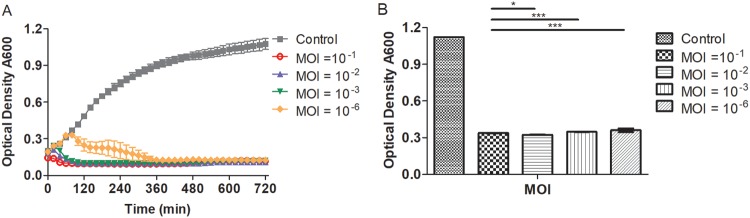
*In vitro* bacterial lytic activities of the phage at various multiplicities of infection (MOIs). (A) Killing curves of a Vibrio alginolyticus strain (GenBank accession no. MH298559) by phage ValSw3-3 at various MOIs (10^−1^, 10^−2^, 10^−3^, and 10^−6^). (B) The final OD_600_ value 12 h after infection at different MOIs. Each point represents the means ± SD from three replicate experiments. Statistical analysis was performed using the Kruskal-Wallis test with the *h* multiple-comparison test. Significant differences were found at the *P < *0.01 level.

The *in vitro* lytic effect of ValSw3-3 on its host bacteria was tested by infecting fresh V. alginolyticus cultures at the exponential phase (optical density at 600 nm [OD_600_] of ∼0.4). As shown in Fig. 2A, the killing curve indicated that ValSw3-3 was highly effective against the host at all MOIs tested in this study, and almost no bacterial growth was observed for the first 12 h after infection. The intense bactericidal activity indicates that ValSw3-3 is a potent candidate for use in *in vivo* phage therapy trials against V. alginolyticus. Generally, the regrowth of the bacterial population following phage infections implies the emergence of phage resistance (15). During our experiment, the regrowth of the bacterial population occurred slowly after infection for 13 h, but the OD_600_ of the culture was below 0.35 even 30 h after infection (Fig. 2B).

Moreover, the killing curves, observed at different MOIs (Fig. 2A), showed no observable difference in inhibitory effects after 5 h of infection, and even the lowest MOI resulted in suppressed host growth throughout the 30 h of the experiment (Fig. 2B). These results suggested that further increasing phage loads would lead to a limited positive effect on the infection of phages with strong lytic ability. Therefore, using heavy phage loads to restrain the emergence of phage resistance in bacteria is not an ideal strategy. Instead, further exploration for cocktails composed of ValSw3-3 and other phages could be a more practical approach to strengthen the antibacterial activity.

### Genomic characteristics of ValSw3-3.

The genome sequence of ValSw3-3 was obtained using an Illumina HiSeq 1500 sequencer platform; approximately 20,000 reads were obtained in total, with an average length of 150 bp. After *de novo* assembly based on overlap of ≥40 nucleotides with a minimum overlap identity of 90%, the whole genome was aligned as a single contig.

**FIG 3:**
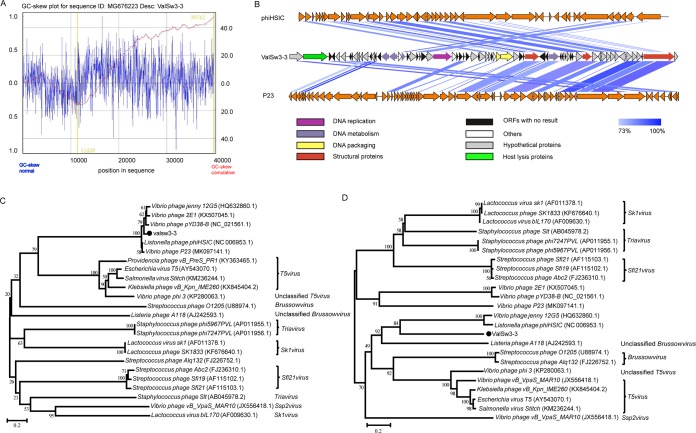
Genomic sequence characterization of ValSw3-3. (A) Cumulative GC skews of phage ValSw3-3. The global minimum and maximum are displayed in the cumulative graph. Putative origin of replication and putative terminus location are highlighted. (B) Multiple-sequence alignment of phage genomes. The whole genomes of *Listonella pelaqia* phage phiHSIC (GenBank accession number NC_006953.1), *Vibrio* phage P23 (MK097141.1), and ValSw3-3 were compared at the DNA level using Easyfig. The blue shading indicates sequence similarities between the genomes. The predicted functions of proteins are indicated by different colors. (C) Phylogenetic trees based on the major capsid proteins (A) and the terminase large subunit (B) of ValSw3-3 and homolog proteins from other phages in the *Siphoviridae* family. Amino acid sequences were aligned using MEGA 5.05 software, and the phylogenetic trees were generated using the neighbor-joining method with 1,000 bootstrap replications. The scale bar represents a phylogenetic distance of 0.2, and the numbers at the nodes represent the percent bootstrap values.

A linear 39,846-bp double-stranded DNA genome with a GC content of 43.1%, similar to that of its host (V. alginolyticus, 44%), was obtained. It has 97 promoter regions and 44 predicted transcription terminators (Table 2). Among the eleven reported *Vibrio* phages belonging to siphovirus (NCBI database as of June 2019), the genome size of phage ValSw3-3 is 2,524 bp larger than the 37,324-bp genome of phage pYD38-B, while its GC content is equal to that of pYD38-B but not other *Vibrio* siphoviruses (Table 2). To identify the origin site of replication and the terminus point of the phage genome, cumulative GC skew was generated as described by Mackiewicz et al. (16). The results indicated that ValSw3-3 had a putative replication origin site at bp 11428 and a putative terminus location at bp 39742 (Fig. 3A), and the GC skew was consistent with the transcriptional directions of most of the open reading frames (ORFs). No tRNA genes were found in the genome of ValSw3-3, indicating that ValSw3-3 depends on the translation machinery of the host (Table 2).

**TABLE 2:** Phage genome characteristics

Phage name	Accession no.	Genome length (bp)	GC content (%)	No. of:				Host
				ORFs	Terminators	Promoters	tRNAs	
ValSw3-3^*a*^	MG676223	39,846	43.1	69	44	97	0	Vibrio alginolyticus
pYD38-B^*b*^	NC_021561.1	37,324	43.1	57	30	88	0	*Vibrio* sp. strain YD38
P23^*b*^	MK097141.1	40,063	42.5	72	44	100	0	Vibrio sagamiensis B23
2E1^*b*^	KX507045.1	40,823	41.7	47	40	99	0	Vibrio anguillarum
pYD21-A^*b*^	NC_020846.1	46,917	43.9	74	38	119	1	*Vibrio* sp. strain YD21
VpKK5^*b*^	NC_026610.1	56,637	51.3	80	57	126	0	Vibrio parahaemolyticus
SHOU24^*b*^	NC_023569.1	77,837	46.0	96	57	181	0	Vibrio parahaemolyticus
vB_VpaS_MAR10^*b*^	NC_019713.1	78,751	49.7	107	55	169	0	Vibrio parahaemolyticus
SIO-2^*b*^	NC_016567.1	81,184	44.9	115	48	195	0	*Vibrio* sp. strain SWAT-3
pVp-1^*b*^	NC_019529.1	111,506	39.7	157	59	274	25	Vibrio parahaemolyticus
phi 3^*b*^	NC_028895.1	116,138	42.8	156	84	279	13	Vibrio cholerae

a Phage isolated in this study.

b *Vibrio* phages within the family *Siphoviridae*; the genome sequences were downloaded from the NCBI database.

Analysis of the phage ValSw3-3 genome revealed 69 putative ORFs. The pVOG database was scanned for homologues of the proteins encoded by the predicted ORFs using BLASTP (E values of less than 10^−4^). Since the genome sequence of ValSw3-3 significantly differed from those of other available phage genomes in NCBI (query coverage, 42%; identity, 88%), only 16% of the ORFs were related to proteins with known or predicted functions, and another 39% had hits of unknown functions. Thirty-eight ORFs showed different levels of similarity (28% to 96%) to their counterparts from 17 species (see Table S1 in the supplemental material) in the BLASTP analysis. Putative functions were assigned to 12 ORFs based on similarities in the amino acid sequences. Despite the low number of annotated ORFs, some representative modules, such as those encoding proteins implicated in DNA replication (helicase), DNA metabolism (endodeoxyribonuclease, RecT recombination, exonuclease, and NAD-asparagine ribosyltransferase), DNA packaging (terminase large/small subunit), structural protein (capsid), and host lysis (lysozyme and hemagglutinin), were recognized in the ValSw3-3 genome (Fig. 3B). These functional modules constitute approximately 40% of the whole-genome length. The remaining ORFs showed less than 28% or no similarity to other proteins in the NCBI database. Pairwise comparison of the genomes of ValSw3-3 and its most closely related phages, phiHSIC (GenBank accession no. NC_006953.1) and P23 (MK097141.1) (17), was carried out. Although most of the ValSw3-3 ORFs (including hypothetical proteins) have no homologs with *Listonella* phage phiHSIC, the terminase large subunit and the major capsid proteins of these two phages share identities of over 85%. Moreover, there were no gene clusters related to lysogeny (e.g., Cro, CI, C2, C3, N, and Q) (18) in the genome, indicating that ValSw3-3 should be considered an obligate lytic bacteriophage that met the prerequisite for phage therapy candidates. Additionally, no antibiotic resistance genes or virulence factor-related genes were detected in the genome of ValSw3-3 using the ARDB (19) and VFDB (20) databases.

### Multiple-genome comparisons, phylogenetic analysis, and comparative genomics.

**FIG 4:**
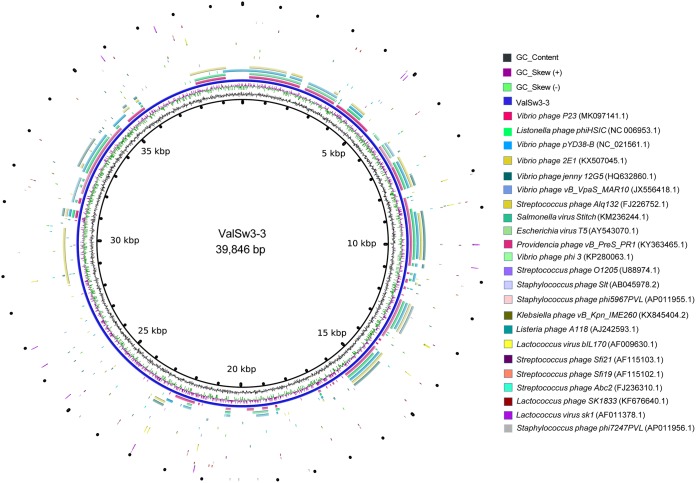
Circular representation depicting the complete genomic comparisons of 23 other phages against ValSw3-3. The GenBank accession numbers of the genomes are listed at the right. The innermost rings of the histogram show GC content (black) and GC skew (green/burgundy) of the ValSw3-3 genome. The third innermost ring, in blue, represents the genome of ValSw3-3. The fragments in different colors outside the blue ring indicate similar regions shared between ValSw3-3 and the other 23 phage genomes based on results from BLAST comparisons. These fragmented rings are arranged from inside out in the same order as the list of the 23 phage genomes. This map was created using BRIG based on the default settings.

**FIG 5:**
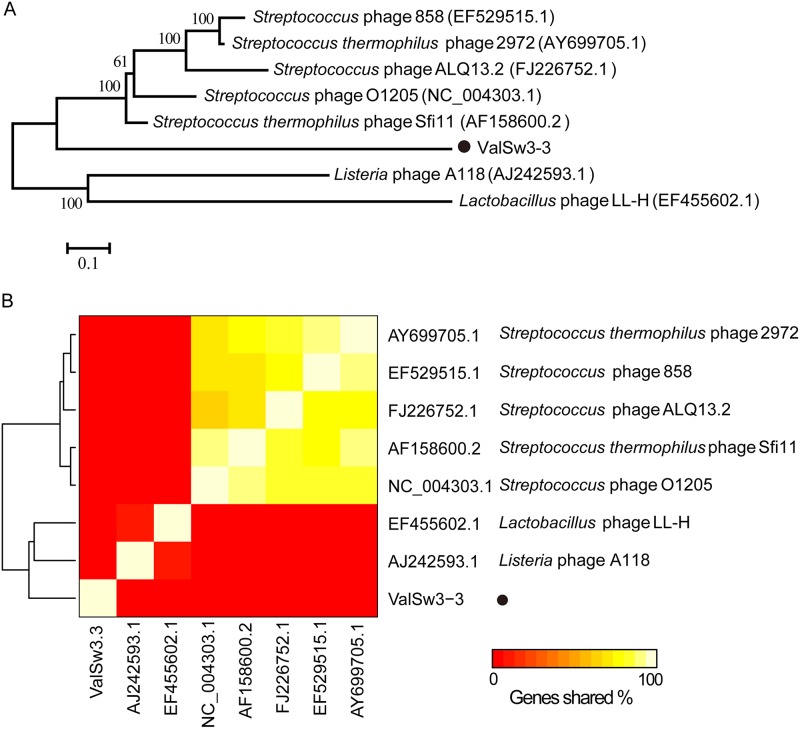
Comparisons of ValSw3-3 with other phages within the *Brussowvirus* genus. (A) Phylogenetic tree based on the whole genome. Shown is a neighbor-joining consensus tree of all known *Brussowvirus* genus phages and ValSw3-3. Numbers next to branches represent consensus support. (B) Heat map showing the percentage of shared genes between phage ValSw3-3 and all known *Brussowvirus* genus phages. Numbers in the boxes indicate phage ValSw3-3 and eight known *sfi11like* phages delineated by this genome comparison.

ValSw3-3 is a siphovirus isolated using V. alginolyticus as the host. To assess the exact taxonomic position of the phage, phylogenetic analysis was performed based on the amino acid sequences of the terminase large subunit (21) and major capsid protein (22) of ValSw3-3 and other reported members of the *Siphoviridae* family. The resulting phylogenetic tree based on the terminase large subunit revealed that ValSw3-3 was most closely related to the five phages, listed as *Listonella* phage phiHSIC (GenBank accession no. NC_006953.1), *Vibrio* phage P23 (MK097141.1), *Vibrio* phage pYD8-B (NC_021561.1), *Vibrio* phage 2E1 (KX507045.1), and *Vibrio* phage 12G5 (HQ632860.1) (Fig. 3C). These six phages formed a branch clearly distinct from other branches corresponding to known genera and ratified by the ICTV. However, according to the phylogenetic tree based on the major capsid protein, ValSw3-3 was only clustered with *Listonella* phage phiHSIC (NC_006953.1) (23, 24) and *Vibrio* phage *jenny* 12G5 (HQ632860.1) (Fig. 3D). To confirm this finding, we carried out comparative analysis of the whole-genome sequences of the same phages used in the phylogenetic analyses. Similar to the results of the major capsid protein analysis, ValSw3-3 grouped with the five phages and shared lower similarity with other phages (Fig. 4). Among the six ratified genera represented in the phylogenetic tree, the branch containing ValSw3-3 was most closely related to the genus *Brussowvirus*. To further clarify the relationship between ValSw3-3 and *Sfi11virus* phages, we performed a phylogenetic analysis (Fig. 5A) and pairwise genomic comparisons (Fig. 5B) between ValSw3-3 and other species within the *Brussowvirus* genus. The results indicated that ValSw3-3 had a distant evolutionary relationship with *Sfi11virus* phages, confirming the novelty of ValSw3-3.

**FIG 6:**
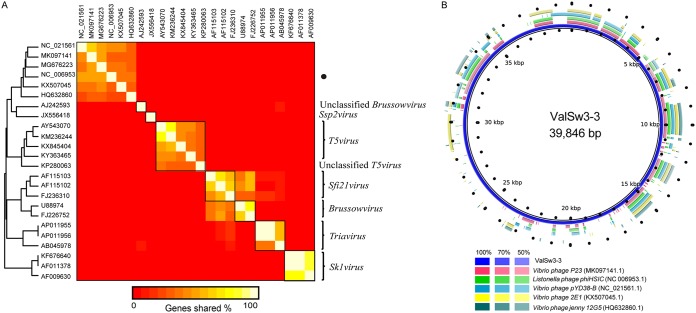
Pairwise comparisons of ValSw3-3 and other phages in the *Siphoviridae* family. (A) *In silico* pairwise comparison of the proteomes of ValSw3-3 and 23 other phages in the *Siphoviridae* family. A proteome is a set of proteins that belong to a species. The sequences were obtained from the annotations of the complete genomes of these phages. ValSw3-3 was marked as a black circle. (B) Genome comparisons of *Listonella* phage phiHSIC (GenBank accession no. NC_006953.1), *Vibrio* phage P23 (MK097141.1), *Vibrio* phage pYD8-B (NC_021561.1), *Vibrio* phage 2E1 (KX507045.1), and *Vibrio* phage 12G5 (HQ632860.1) against ValSw3-3. The various color gradients of rings 1 to 6 indicate the BLAST match of a particular percent identity, as shown in the rectangles below. This map was created using BRIG based on default settings.

All five phages clustered with ValSw3-3 in the phylogenetic analysis are siphoviruses, but none of them have been classified at the genus level to date. Therefore, to facilitate a more detailed classification, we conducted an *in silico* pairwise comparison of the proteomes of ValSw3-3 and the five phages, as well as 18 other distantly related phages that were well classified and ratified by the ICTV. This analysis revealed five similarity groups (Fig. 6A). Typically, phages within a genus are defined as sharing more than 35% of their genes (25, 26). The ratios of shared genes between ValSw3-3 and *Vibrio* phage P23 (GenBank accession no. MK097141.1), *Vibrio* phage pYD8-B (NC_021561.1), and *Vibrio* phage 2E1 (KX507045.1) were above the threshold value, namely, 52.2% (36/69), 39.1% (27/69), and 36.2% (25/69). Although ValSw3-3 shared only 34.8% (24/69) and 30.4% (21/69) of its homologous genes (amino acid identity, 69% on average) with phiHSIC (NC_006953.1) and 12G5 (HQ632860.1), respectively, these three phages were grouped together in both phylogenetic trees. Overall, the results of proteome comparisons were consistent with the results of phylogenetic analysis. Complete genome sequence comparisons of the five phages *Listonella* phage phiHSIC (NC_006953.1), *Vibrio* phage P23 (MK097141.1), *Vibrio* phage pYD8-B (NC_021561.1), *Vibrio* phage 2E1 (KX507045.1), and *Vibrio* phage 12G5 (HQ632860.1) with ValSw3-3 further confirmed different similarity regions located throughout the phage genome, and the most similar regions were from bp 6500 to 7000, bp 8800 to 10500, and bp 14300 bp to 15800 (Fig. 6B). DNA sequence identity is an essential parameter for identifying new members of the viruses classified by the ICTV, and those with genome sequence identity of less than 50% can be defined as a new genus (27). Given that the highest sequence query coverage was merely 42%, observed when the genome of phage phiHSIC was used as the reference, our analyses allow us to propose a novel genus to accommodate phage ValSw3-3 in the family *Siphoviridae* (an application has been submitted to the ICTV).

### Core gene analysis.

**FIG 7:**
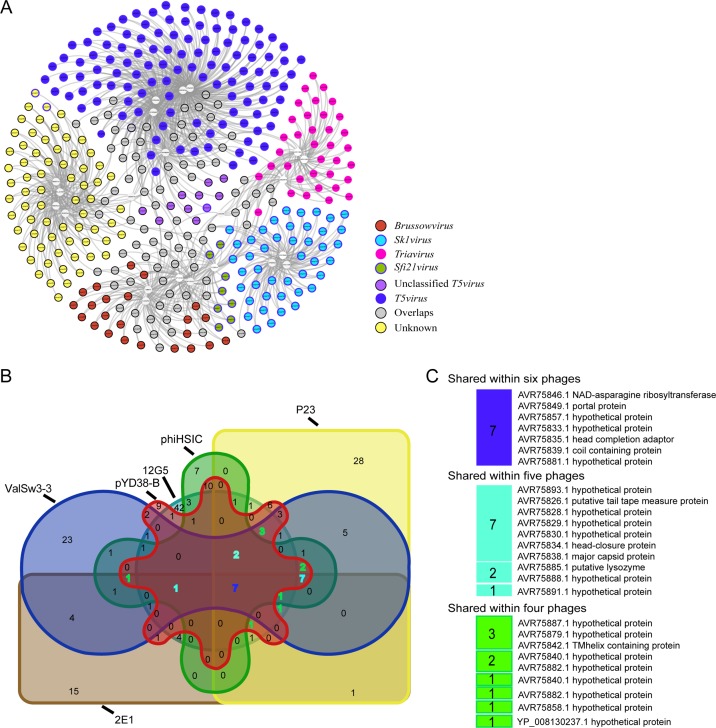
Orthologous analysis and shared core gene analysis. (A) A network of shared ortholog clusters of the predicted proteins. White nodes represent all 24 phage genomes used for the core gene search. Other colored nodes connected to the white nodes by lines represent ortholog clusters generated by multiple-sequence alignments of all the proteins obtained from the gene annotation of the 24 phage genomes. Red nodes indicate the protein clusters shared within genus *Brussowvirus*, cyan nodes within genus *Sk1virus*, ruby nodes within genus *Triavirus*, olive nodes within genus *Sfi21virus*, plum nodes within genus Unclassified *T5virus*, and blue nodes within genus *T5virus*, while gray nodes represent the clusters shared across genera. Moreover, yellow nodes represent clusters shared by phiHSIC (GenBank accession no. NC_006953.1), P23 (MK097141.1), pYD8-B (NC_021561.1), 2E1 (KX507045.1), 12G5 (HQ632860.1), and ValSw3-3. (B) The shared core proteins shown in panel A among phages phiHSIC (NC_006953.1), P23 (MK097141.1), pYD8-B (NC_021561.1), 2E1 (KX507045.1), 12G5 (HQ632860.1), and ValSw3-3 are visualized in the Venn diagram. (C) The predicted functions of the proteins shared by more than three phages are listed in color columns together with their accession numbers. The colors of the columns and the numbers highlighted in panel B are consistent.

Putative ortholog analysis was performed with a set containing all the proteins predicted from the 24 phage (ValSw3-3, P23, 2E1, phiHSIC, pYD8-B, 12G5, and 18 other distantly related phages) genomes to screen for core genes. A total of 2,500 protein sequences from the 24 phage genomes were grouped into 384 clusters. As expected, due to phage diversity, no cluster contained genes from all phage genomes (Fig. 7A). Therefore, we identified the genes in each ortholog group and determined the numbers of shared core clusters among groups. The results showed that the core genes among most clusters were annotated as terminase large subunit, major capsid protein, tail protein, and DNA polymerase. This reflected the conservative nature of the genes encoding these proteins and corroborated the feasibility of phylogenetic analyses of these phages based on these proteins. The results also revealed large numbers of hypothetical proteins shared within each of the clusters, which suggested that very little has been done to functionally characterize phage proteins, even though many of the phages with their genomes sequenced may have great potential in biomedical applications. In total, the ValSw3-3 genome contained 69 clusters, among which 47 were shared with phiHSIC, P23, pYD8-B, 2E1, or 12G5 (Fig. 7B). Seven clusters commonly shared with phiHSIC, P23, pYD8-B, 2E1, and 12G5 were annotated as a portal protein, NAD-asparagine ribosyltransferase head completion adaptor, coil-containing protein, and three hypothetical proteins (Fig. 7C). Interestingly, the two clusters (portal protein and NAD-asparagine ribosyltransferase) of ValSw3-3 were also shared with vB_VpaS_MAR10 (GenBank accession no. JX556418.1), a temperate Vibrio parahaemolyticus phage. Furthermore, a sequence similarity search of the ORF of NAD-asparagine ribosyltransferase in UniProt or the NCBI database revealed that this protein was encoded only by *Vibrio* phage genomes, except for *Listonella* phiHSIC. It should be noted that Listonella pelagia is not found to be distinct from the *Vibrio* genus based on the genomic and phenotypic data, and members of this taxon were formerly named *Vibrio pelagia* (23, 28). As for the other shared clusters in the ValSw3-3 genome, there were ten clusters shared with four out of the five phages and nine clusters with three. Clusters annotated as major capsid protein, tail tape measure protein, head closure protein, and four hypothetical proteins were shared with phiHSIC, P23, pYD8-B, and 2E1, while putative lysozyme was shared with 12G5 but not 2E1. Structural proteins, such as the major capsid protein (29), tail tape measure protein (30), head protein (31), and terminase large subunit (32), are widely used for phage phylogenetic analysis due to their conserved sequences. Notably, the terminase large subunit of ValSw3-3 was not included in the commonly shared clusters by phiHSIC, P23, pYD8-B, 2E1, 12G5, and ValSw3-3 but only grouped with phiHSIC and 12G5. This finding suggested that it is rational to use various strategies when assessing the taxonomy of phages. Lysozymes (endolysins) are highly diverse proteins both enzymatically and structurally and now are one of the most promising families of alternative antimicrobials (33, 34).

### Conclusions.

In summary, this study demonstrates that ValSw3-3, isolated using a V. alginolyticus strain (GenBank accession no. MH298559) as the host, is a novel lytic phage of the family *Siphoviridae*. As characterized by determining its host range, one-step growth curve, and lytic activity, ValSw3-3 exhibits strong lytic activity and great potential as a novel antibacterial agent for the biological control of vibriosis in aquaculture. Moreover, ValSw3-3 is stable at up to 50°C and pH ranging from 4 to 10, which makes it suitable for potential use in seawater environments. Phylogenetic analyses and core gene analysis suggest that a new viral genus can be proposed to accommodate ValSw3-3. Although a large portion of clusters still are annotated as hypothetical proteins, several relatively conserved genes were identified by the analysis of core genes, including those encoding terminase large subunit, capsid protein, and tail protein. Furthermore, a unique gene encoding NAD-asparagine ribosyltransferase in the ValSw3-3 genome was found to be of specificity in the core gene analysis, and it could serve as a marker gene of *Vibrio* phages. For further understanding of ValSw3-3, our work will focus on the functional characterizations needed to enable biomedical applications of this phage in various contexts.

## MATERIALS AND METHODS

### Isolation and identification of bacterial pathogens.

Bacterial strains used in this work were isolated from diseased shrimps and water in different aquafarms (Shenzhen, Guangdong province, China; Shanwei, Guangdong province, China; and Wuhan, Hubei province, China). *Vibrio* strains were isolated using thiosulfate citrate bile salt-sucrose agar medium (TCBS agar; Haibo, China) and further incubated at ambient temperature (28°C) in 2216E medium (5 g peptone and 2 g yeast extract per liter). Taxonomy assignment of these strains was achieved by 16S rRNA gene sequence analysis (35). Briefly, microbial DNA samples were used as templates for the amplification of 16S rRNA gene fragments using the universal primers 27f and 1492r. The reaction mixture consists of 10 ng of template DNA, 2.5 U of DNA polymerase, 5 μl of 10× PCR amplification buffer (100 mM Tris-HCl, 500 mM KCl), 200 μM deoxynucleoside triphosphates, 1.5 mM MgCl_2_, and 10 pmol of a primer. The mixture described above first was denatured for 1 min at 98°C, followed by 30 PCR cycles of denaturation (10 s at 98°C), annealing (30 s at 56°C), and extension (40 s at 72°C). Finally, another extension was executed at 72°C for 2 min. The PCR products were sequenced by a Sanger dideoxy sequence platform (BGI Gene Co., Ltd., China).

### Phage isolation, purification, and host range determination.

One of the bacterial isolates, V. alginolyticus strain Va-F4 (GenBank accession no. MH298559), caused high mortality rates (53.1%) of shrimps during the challenge test in our previous study (36). Using this strain as the host bacterium, phage ValSw3-3 was isolated from sewage from an aquaculture farm in Shanwei, China. The isolation step was performed as described previously (37, 38).

The host range of ValSw3-3 was determined by spot testing and confirmed by the double-layer agar method. Briefly, the test strains listed in Table 1 were individually cultured to an OD_600_ of about 0.5, an aliquot of 350 μl of each culture was individually added to 5 ml of prewarmed semisolid liquid medium, and then the ingredients were mixed and poured onto individual plates containing solid medium to form a double-layer plate. Ten microliters of the purified phage solution (10^11^ PFU/ml) was dropped onto the overlaid top agar. The plates then were incubated at 30°C and examined for the presence of a lysis zone after 12 h.

### Transmission electron microscopy.

Purified phage particles from a highly concentrated phage lysate (10^10^ PFU/ml) in SM buffer (100 mM NaCl, 8 mM MgSO_4_, 5 mM Tris-HCl [pH 7.5], 0.01% gelatin) were adsorbed onto carbon-coated copper grids and then negatively stained with 2% phosphotungstic acid (pH 7.0). After drying at room temperature, the grids were examined using a TECNAI G2 F20 S-Twin transmission electron microscope (FEI, USA).

### One-step growth curve.

To determine the one-step growth curves of the phage, a modification of the method of Sasikala and Srinivasan (1) was used. In brief, 6 ml of V. alginolyticus (GenBank accession no. MH298559) cells (OD_600_ of 0.5 to 0.6) was centrifuged at 8,000 rpm for 5 min. The cell pellets were resuspended in 300 μl of 2216E liquid medium, and then 600 μl of phage suspension was added to yield a multiplicity of infection (MOI) of 0.01. After adsorption for 10 min at 30°C, the mixtures were centrifuged at 12,000 rpm for 10 min to remove any unadsorbed phage particles prior to resuspending the cell pellets in 6 ml of 2216E liquid medium. The samples then were shaken and incubated at 30°C, and aliquots were collected at different intervals over a 60-min period. These aliquots were immediately diluted, and phage titers were determined using the double-layer agar method (39). Three independent experiments were performed for each assay.

### pH and thermal stability assays.

Environmental factors, including pH and temperature, were tested according to the methods described by Kim et al. (10). For pH stability tests, 100 μl filter-sterilized phage samples (10^8^ PFU/ml) was inoculated in 1 ml SM buffer (100 mM NaCl, 8 mM MgSO_4_, 5 mM Tris-HCl, 0.01% gelatin, pH 7.5), adjusted to pH 2 to 12 with 1 M NaOH and 1 M HCl, and incubated at 30°C for 1 h statically. For thermal stability tests, 1 ml filter-sterilized phage samples (10^8^ PFU/ml) was incubated statically under the indicated condition (4°C, 10°C, 20°C, 30°C, 37°C, 42°C, 50°C, and 65°C) for 1 h. After the treatment, aliquots were collected and assayed to determine the number of surviving plaque-forming units.

### Growth curve experiment.

An *in vitro* lysis assay for phage ValSw3-3 on V. alginolyticus (GenBank accession no. MH298559) was determined at various MOIs (10^−6^, 10^−3^, 10^−2^, and 10^−1^). The measurement was performed in sterile 100-well plates using a growth curve measuring instrument (110001-892; BioScreen C; Growth Curves USA) by monitoring the OD_600_ (optical density measurements at a wavelength of 600 nm). Briefly, wells were loaded with 200 μl of freshly prepared culture of the host bacteria. The plate was placed in the instrument and incubated at 30°C with orbital shaking. The initial purified phage stock solution was prepared, and the phage titers were adjusted to desired concentrations by serial dilution. Cultures were infected with equal volumes of phage lysate at 4 different MOIs when the bacterial culture reached the exponential phase (OD_600_ of ∼0.4). For each assay, two control samples were included: the bacterium-only control, which was inoculated with V. alginolyticus (MH298559) only, and the phage control, which was inoculated with the phage lysate without bacteria. The growth curves were monitored in real time over 30 h, and OD_600_ measurements were recorded every 5 min. For all phage infection assays, the titer of each initial phage inoculum had been determined previously using the double agar layer method. Three independent assays were carried out for each assay.

### Phage DNA extraction, genome sequencing, and assembly.

The concentrated phage particles were treated using DNase I and RNase A (New England BioLabs) to remove bacterial nucleic acids. The phage genomic DNA then was extracted using a lambda bacteriophage genomic DNA rapid extraction kit (DN22; Aidlab, China) by following the manufacturer’s protocol and sequenced using an Illumina HiSeq 1500 sequencer platform (Annoroad Gene Technology Co., Ltd., China). The high-quality filtered reads were assembled using SOAP *de novo* with default parameters (40). The complete genome sequence then was manually inspected.

### Genome analysis, phylogenetic analysis, and core gene analysis.

Open reading frames (ORFs) were predicted with GeneMark.hmm (http://exon.gatech.edu/GeneMark/index.html) (41, 42). The ORFs were annotated using the BLASTP algorithm with the nonredundant (nr) protein database of the National Center for Biotechnology Information (NCBI) (https://www.ncbi.nlm.nih.gov), with an E value cutoff of 10^−4^. The visualization of comparisons of multiple phage genome sequences was performed using BLAST Ring Image Generator (BRIG) with default settings (43). The putative promoter sequences were predicted using the online tools Softberry (http://www.softberry.com/berry.phtml) (44) and Promoter Scan (https://www-bimas.cit.nih.gov/molbio/proscan). The presence of virulence genes was searched with the Virulence Search program (http://www.hpa-bioinfotools.org.uk/pise/virfactfind_small.html). Rho-independent transcription terminators were also predicted using the ARNold program (http://rna.igmors.u-psud.fr/toolbox/arnold). tRNAs carried by the phage genomes were detected using the protein ARAGORN and tRNAscan-SE (http://lowelab.ucsc.edu/cgi-bin/tRNAscan-SE2.cgi) (45). Comparative genomic analysis was conducted using EasyFig 2.1. (46). To determine the taxonomy of the isolated phages, phylogenetic analysis based on the terminase large subunit was carried out using MEGA 5.02 software (http://www.megasoftware.net) with the neighbor-joining method and 1,000 bootstrap replications (47). Pairwise similarity comparisons of each predicted protein were performed using BLASTP and visualized using the R, version 3.4.1, gplots package (48). Furthermore, core gene analysis was carried out with the established pipeline orthoMCL (49) (https://orthomcl.org/orthomcl/), and the resulting network was visualized with Cytoscape software (version 3.6.1; http://cytoscape.org/), using an edge-weighted spring-embedded model, which places the phage genomes that share more protein clusters (which clusters proteins based on similarity) in closer proximity in the display. Moreover, the proteins shared by the target phage (ValSw3-3) with others were also visualized in the Venn diagram constructed with the aid of the online software DrawVenn (http://bioinformatics.psb.ugent.be/webtools/Venn/).

### Statistical analysis.

The SPSS software package, version 12.0, was used for all statistical analyses. Statistically significant differences in all of the experiments were determined using the Kruskal-Wallis test with the *h* multiple-comparison test at *P *values of <0.01.

### Data availability.

The complete genome sequence of the phage ValSw3-3 is available in GenBank under accession number MG676223. The 16S rRNA gene sequences are also available in the NCBI GenBank database under the accession numbers listed in Table 1.

## Supplementary Material

Supplemental file 1
