# Supplementary material for: Characterization and Genomic Analysis of ValSw3-3, a New Siphoviridae Bacteriophage Infecting Vibrio alginolyticus
Source: J Virol. 2020 May 4;94(10):e00066-20. doi: 10.1128/JVI.00066-20 (PMC7199398; doi:10.1128/JVI.00066-20)
Supplement: Supplemental file 1 [file JVI.00066-20-s0001.pdf]

Supplemental Table

Table S1 Features of ORFs, gene products and putative functions of phage ValSw3-3

ORF	Position		Length (aa)	Accession number	Description		
	From	To			Closest hit in NCBI	Similarity (%)	E-value
ORF_1	2	343	113	AVR75825.1	hypothetical protein [Listonella phage phiHSIC]	80.0	1.80E-06
ORF_2	343	3531	1062	AVR75826.1	putative tail tape measure protein [Listonella phage phiHSIC]	70.2	0.00E+00
ORF_3	3630	3764	44	AVR75827.1	-		
ORF_4	3780	4208	142	AVR75828.1	hypothetical protein [Listonella phage phiHSIC]	44.4	3.70E-25
ORF_5	4220	4702	160	AVR75829.1	hypothetical protein [Listonella phage phiHSIC]	90.6	4.60E-77
ORF_6	4771	5241	156	AVR75830.1	hypothetical protein [Listonella phage phiHSIC]	35.6	3.60E-10
ORF_7	5362	6036	224	AVR75831.1	hypothetical protein [Listonella phage phiHSIC]	56.4	2.60E-33
ORF_8	6063	6458	131	AVR75832.1	hypothetical protein [Listonella phage phiHSIC]	59.8	1.50E-33
ORF_9	6460	6930	156	AVR75833.1	hypothetical phage protein [Listonella phage phiHSIC]	56.4	1.50E-43
ORF_10	6935	7336	133	AVR75834.1	hypothetical phage protein [Listonella phage phiHSIC]	89.3	2.70E-62
ORF_11	7340	7837	165	AVR75835.1	hypothetical phage protein [Listonella phage phiHSIC]	87.9	3.00E-79
ORF_12	7922	8197	91	AVR75836.1	hypothetical protein S140_58 [Shewanella sp. phage 1/40]	60.2	1.20E-24
ORF_13	8235	8789	184	AVR75837.1	hypothetical protein [Listonella phage phiHSIC]	58.0	1.90E-50
ORF_14	8851	9795	314	AVR75838.1	major capsid protein [Listonella phage phiHSIC]	96.2	7.20E-167
ORF_15	9807	10457	216	AVR75839.1	hypothetical protein [Listonella phage phiHSIC]	85.2	5.80E-83
ORF_16	10706	10885	59	AVR75840.1	-		
ORF_17	10869	11075	68	AVR75841.1	-		
ORF_18	11072	11386	104	AVR75842.1	hypothetical protein [Listonella phage phiHSIC]	59.3	2.50E-23
ORF_19	11465	11620	51	AVR75843.1	-		
ORF_20	11750	11971	73	AVR75844.1	-		
ORF_21	11968	12147	59	AVR75845.1	-		
ORF_22	12176	13246	356	AVR75846.1	putative NAD-asparagine	89.3	3.10E-17

					ribosyltransferase [Listonella phage phiHSIC]		4
ORF_23	13321	13608	95	AVR75847.1	-		
ORF_24	13598	14233	211	AVR75848.1	-		
ORF_25	14262	15692	476	AVR75849.1	putative structural protein [Listonella phage phiHSIC]	85.5	7.90E-242
ORF_26	15753	15893	46	AVR75850.1	-		
ORF_27	15893	16291	132	AVR75851.1	-		
ORF_28	16291	16857	188	AVR75852.1	hypothetical protein TUST1-2_01130 [Vibrio phage ICP1_2001_A]	36.8	7.00E-08
ORF_29	16927	18186	419	AVR75853.1	phage terminase, large subunit, PBSX family [Thermoanaerobacterium phage THSA-485A]	45.1	1.70E-86
ORF_30	18170	18610	146	AVR75854.1	phage terminase small subunit [Enterobacteria phage CUS-3]	58.1	1.30E-40
ORF_31	18689	18973	94	AVR75855.1	hypothetical protein CcrMagneto_gp158 [Caulobacter virus Magneto]	41.9	4.70E-21
ORF_32	18970	19182	70	AVR75856.1	-		
ORF_33	19179	19421	80	AVR75857.1	hypothetical protein [Listonella phage phiHSIC]	43.8	2.90E-11
ORF_34	19418	19765	115	AVR75858.1	hypothetical protein [Listonella phage phiHSIC]	58.3	1.90E-32
ORF_35	19762	20367	201	AVR75859.1	hypothetical protein Phi18:2_gp02 [Cellulophaga phage phi18:2]	47.4	1.80E-41
ORF_36	20364	20516	50	AVR75860.1	-		
ORF_37	20582	21256	224	AVR75861.1	hypothetical protein S-CBP4_0046 [Synechococcus phage S-CBP4]	30.3	
ORF_38	21400	21762	120	AVR75862.1	-		
ORF_39	21904	22047	47	AVR75863.1	-		
ORF_40	22114	22467	117	AVR75864.1	-		
ORF_41	22536	22742	68	AVR75865.1	-		
ORF_42	22751	23179	142	AVR75866.1	PRK09741 superfamily protein [Enterobacteria phage phi80]	28.7	2.80E-17
ORF_43	23221	25095	624	AVR75867.1	DNA primase/helicase [Salmonella phage vB_SosS_Oslo]	58.5	1.60E-218
ORF_44	25092	25823	243	AVR75868.1	hypothetical protein BA3_0036 [Thalassomonas phage BA3]	33.5	1.60E-17

ORF_45	25826	26344	172	AVR75869.1	-		
ORF_46	26400	26534	44	AVR75870.1	-		
ORF_47	26623	26841	72	AVR75871.1	-		
ORF_48	26838	27596	252	AVR75872.1	hypothetical protein [Enterobacteria phage phiP27]	42.0	1.10E-29
ORF_49	27599	27826	75	AVR75873.1	-		
ORF_50	27973	28350	125	AVR75874.1	crossover junction endodeoxyribonuclease [Erwinia phage phiEt88]	37.8	1.70E-13
ORF_51	28343	28693	116	AVR75875.1	hypothetical protein SfIV_45 [Shigella phage SfIV]	42.5	1.20E-05
ORF_52	28810	29655	281	AVR75876.1	putative RecT recombination protein [Aeromonas phage vB_AsaM-56]	64.4	2.90E-98
ORF_53	29652	30467	271	AVR75877.1	putative exonuclease [Aeromonas phage vB_AsaM-56]	53.7	4.70E-74
ORF_54	30470	30835	121	AVR75878.1	-		
ORF_55	30956	31141	61	AVR75879.1	-		
ORF_56	31134	31436	100	AVR75880.1	hypothetical protein S349_53 [Shewanella sp. phage 3/49]	35.9	6.60E-05
ORF_57	31429	31638	69	AVR75881.1	-		
ORF_58	31810	32346	178	AVR75882.1	hypothetical protein [Listonella phage phiHSIC]	52.0	8.00E-46
ORF_59	32355	32594	79	AVR75883.1	hypothetical protein S349_19 [Shewanella sp. phage 3/49]	47.4	8.60E-08
ORF_60	32686	32862	58	AVR75884.1	-		
ORF_61	32862	33320	152	AVR75885.1	putative phage lysozyme [Listonella phage phiHSIC]	91.5	3.40E-77
ORF_62	33384	33536	50	AVR75886.1	-		
ORF_63	33536	33742	68	AVR75887.1	-		
ORF_64	34120	34578	152	AVR75888.1	hypothetical protein [Listonella phage phiHSIC]	36.1	1.10E-16
ORF_65	34731	35102	123	AVR75889.1	hypothetical protein SS1_28 [Cronobacter phage vB_CsaP_Ss1]	43.9	1.20E-16
ORF_66	35204	35524	106	AVR75890.1	-		
ORF_67	35499	35825	108	AVR75891.1	-		
ORF_68	35978	38437	819	AVR75892.1	putative hemagglutinin protein [Listonella phage phiHSIC]	87.5	3.60E-40
ORF_69	38490	39846	451	AVR75893.1	hypothetical protein [Listonella phage phiHSIC]	82.2	2.90E-129

3 “-“: genes which have no hit in NCBI database with 1E-4 as the e-value cutoff.
